# ZnO Quantum Photoinitiators as an All-in-One Solution for Multifunctional Photopolymer Nanocomposites

**DOI:** 10.1021/acsnano.3c06518

**Published:** 2023-10-03

**Authors:** Tom Naor, Shira Gigi, Nir Waiskopf, Gila Jacobi, Sivan Shoshani, Doron Kam, Shlomo Magdassi, Ehud Banin, Uri Banin

**Affiliations:** † The Institute of Chemistry and The Center for Nanoscience and Nanotechnology, The Hebrew University of Jerusalem, Jerusalem 91904, Israel; ‡ The Mina and Everard Goodman Faculty of Life Sciences and Advanced Materials and Nanotechnology Institute, Bar-Ilan University, Ramat-Gan 5290002, Israel; § Casali Center for Applied Chemistry, Institute of Chemistry, The Hebrew University of Jerusalem, 91904 Jerusalem, Israel

**Keywords:** nanocomposites, ZnO, photopolymerization, 3D printing, antimicrobial, photocatalysis

## Abstract

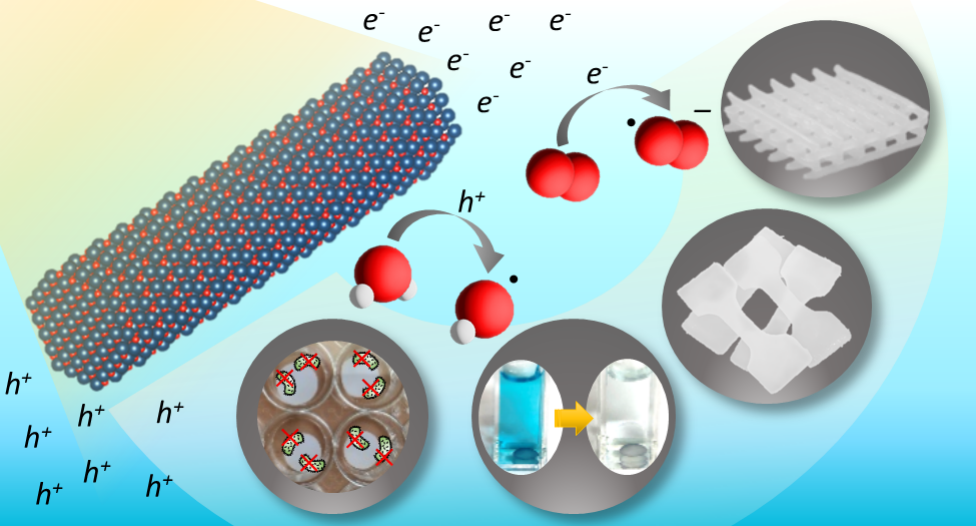

Nanocomposites are constructed from a matrix material combined with dispersed nanosized filler particles. Such a combination yields a powerful ability to tailor the desired mechanical, optical, electrical, thermodynamic, and antimicrobial material properties. Colloidal semiconductor nanocrystals (SCNCs) are exciting potential fillers, as they display size-, shape-, and composition-controlled properties and are easily embedded in diverse matrices. Here we present their role as quantum photoinitiators (QPIs) in acrylate-based polymer, where they act as a catalytic radical initiator and endow the system with mechanical, photocatalytic, and antimicrobial properties. By utilizing ZnO nanorods (NRs) as QPIs, we were able to increase the tensile strength and elongation at break of poly(ethylene glycol) diacrylate (PEGDA) hydrogels by up to 85%, unlike the use of the same ZnO NRs acting merely as fillers. Simultaneously, we endowed the PEGDA hydrogels with post-polymerization photocatalytic and antimicrobial activities and showed their ability to decompose methylene blue and significantly eradicate antibiotic-resistant bacteria and viral pathogens. Moreover, we demonstrate two fabrication showcase methods, traditional molding and digital light processing printing, that can yield hydrogels with complex architectures. These results position SCNC-based systems as promising candidates to act as all-in-one photoinitiators and fillers in nanocomposites for diverse biomedical applications, where specific and purpose-oriented characteristics are required.

## Introduction

Polymer nanocomposites are constructed from a continuous polymeric matrix material combined with a nanosized property-modifying filler. This diverse and flexible combination yields the powerful ability to tailor the desired mechanical, optical, electrical, physical, and antimicrobial material properties.^1−7^ Colloidal semiconductor nanocrystals (SCNCs) are very interesting fillers, as they showcase size-, shape-, and composition-controlled properties and can be easily embedded in a variety of matrices.^8−11^ While their optical functionality has already been addressed in numerous studies and brought to real-life applications,^8,12−14^ the utilization and multifunctional outcomes of photocatalytic SCNCs in such nanocomposites have not yet been sufficiently addressed.^4,15,16^ Herein we investigate the multifunctionality of ZnO nanocrystals as a model for photocatalytic SCNCs acting as photoinitiators in acrylate-based polymers, where they function as a photocatalytic radical initiator and endow the system with mechanical, photocatalytic, and antimicrobial properties.

At the base of this work is the utilization of SCNCs as photoinitiators, termed quantum photoinitiators (QPIs).^17^ Early works reported the use of ZnO or TiO_2_ powders to initiate photopolymerization.^18−20^ This idea has gained momentum more recently, based upon tremendous developments in controlling the size, morphology, and surface chemistry of SCNCs.^5,21−24^ The high light sensitivity of QPIs and their spectral tunability, driven by quantum confinement, result in the manifestation of nearly 3 orders of magnitude higher absorptivity than a typical organic photoinitiator.^17^ This lends them enhanced photocatalytic activity within their broad excitation window and enables their use as photoinitiators suitable for a wide range of light sources.^8^ This is particularly relevant in the field of additive manufacturing, in which digital light processing (DLP) is a key method for 3D printing via photopolymerization. It employs a digital micromirror device to sequentially photopolymerize individual object layers with precise thickness control. First, the desired layer is selectively solidified in a single exposure by projecting a 2D pattern onto a liquid photocurable resin using UV or visible light. Then, the built platform is elevated to replenish the printing area with uncured resin, creating subsequent layers. This process is repeated until the fabrication is fully completed. In the case of 3D printing of aqueous formulations in particular, there is a need for more efficient biocompatible photoinitiators.^25^ QPIs address this gap based on the combination of their unique photophysical and photochemical properties along with their flexible surface chemistry allowing for their dispersion and activity in polar solvent formulations.^21,22^

Moreover, QPIs function via a mechanism different from that of organic photoinitiators. The latter are mostly consumed during the curing process while leaving harmful byproducts, whereas QPIs are not consumed and generate radicals in a photocatalytic cycle, resulting in a different polymerization outcome.^17,26,27^ This raises an important question regarding the characteristics of polymer nanocomposites in which SCNCs are not just fillers but also act as the QPIs. This can yield polymer branching and cross-linking that may differ significantly from the photopolymerization outcome with organic photoinitiators. In turn, this can affect the mechanical properties of the system.

In prior research conducted by Zhang et al., hydrogels were prepared by irradiation of dispersions containing *N*,*N*-dimethyl acrylamide as the monomers, TiO_2_ and ZnO as photoinitiators, and clay nanosheets as reinforcement building blocks.^5^ Mechanical properties were studied with a focus on the effect of the clay nanosheets. A separate analysis of the effect of the QPI action versus the use of the SCNCs merely as fillers on the mechanical properties has thus not yet been addressed. In this elegant prior work, additional post-polymerization photocatalytic functionality of dye degradation by TiO_2_ was also demonstrated. The main effect was the adsorption of the dye into the hydrogel, followed by degradation of the dye in the dried hydrogel disc under UV illumination. Cadmium-based SCNCs were used to show another post-polymerization activity of QPIs, by utilizing their fluorescence ability to create light-emitting 3D-printed nanocomposites.^21^ However, the toxicity of heavy-metal nanocrystals prevents them from being used as QPIs or fillers in nanocomposites for important environmentally friendly solutions such as water-purification filter systems and bioprinting applications.

Another exciting possible contribution of QPIs is the utilization of the antibacterial activity of SCNCs in nanocomposites for biomedical solutions, photocoatings, and filter systems. The ability of bacteria to colonize abiotic surfaces and induce contamination and infection in the medical setting is of great concern. Additionally, avoiding pathogenic microorganism contamination in the water industry and during food manufacturing, processing, and packaging is one of the industry’s major challenges with considerable importance to public health. Thus, the ability to provide effective, safe, and affordable antibacterial coatings is of great value, especially in light of the increase in antimicrobial resistance observed over the last two decades. Subsequent global concern over this worrying trend, along with additional bacterial and viral threats, has created a demand for efficient antimicrobial agents. Examples of solutions utilized heretofore include Ag nanoparticles (NPs), quaternized amines, and metal oxides, among others, which have a wide antibacterial spectrum.^4,28^ Ag NPs, in fact, possess one of the highest levels of antibacterial activity in biomedical applications. However, the drawback of Ag NPs is the continuous dissolution and formation of Ag^+^, which is toxic to biological systems, even at very low concentrations.^29^ Therefore, the high stability and relative nontoxicity of metal oxides, such as TiO_2_, CuO, Fe_2_O_3_, ZnO, and others, have made them the subject of extensive study as efficient bactericidal agents.^30^ Of special note is ZnO, the high biocompatibility and low toxicity of which has made it a preferred antibacterial agent as compared with other metal oxides.^31^ The nonharmful nature of ZnO is evidenced by its listing as generally recognized as safe (GRAS) by the United States Federal Drug Administration (21CFR182.8991). Several mechanisms have been linked to the antibacterial activity of ZnO, including the generation of reactive oxygen species (ROS), membrane dysfunction upon direct contact by electrostatic interactions, and accumulation of zinc ions, which interfere with respiratory processes inside the bacterial cell.^32,33^ The antibacterial activity of ZnO has been suggested to be greatly affected by its crystal morphology, structure, and size. Nanoscale particles of ZnO have shown a greater biocidal effect than bulk ZnO.^34^ ZnO has additional advantages including it being highly abundant, cheap, biocompatible, and commonly used in daily life products, such as sunscreens, cosmetics, and toothpastes.^35,36^ Moreover, it is resistant to oxidation,^37^ providing stability in water and under oxidative conditions.

In this work, we report the use of ZnO nanorods (NRs) as an all-in-one QPI system. Rod-shaped nanocrystals were chosen as a model system, due to their favorable charge separation and photocatalytic activity over nanosized pyramids, attributed to their anisotropic morphology and relatively large, highly reactive zinc-rich facet, respectively.^24^ First, we show their capacity to photopolymerize water-based poly(ethylene glycol) diacrylate (PEGDA) hydrogels. We further show that our ZnO NRs can also be efficiently applied as QPIs in digital light processing (DLP) 3D printing, which provides the important ability to easily realize photopolymerized hydrogels in numerous desired structures. Following our success in fabricating both molded and 3D-printed QPI-based photopolymer nanocomposites, a detailed study of the mechanical properties is reported herein, specifically comparing the characteristics of the systems in which rod-shaped ZnO nanocrystals act as QPI fillers versus their action merely as fillers while utilizing organic photoinitiators for polymer curing. Finally, post-polymerization multifunctionality is also studied, demonstrating the dye degradation effect *in situ* and ROS formation. Special attention is given to the antimicrobial function of the formed objects, revealing the ability to efficiently reduce biofilm formation and eradicate planktonic bacteria, even of multi-drug-resistant strains. This work presents a photocatalytic and antimicrobial active photopolymeric nanocomposite that requires no harmful organic photoinitiators, no additional reinforcement agents, and no addition of antimicrobial agents, since the ZnO NRs acting as QPIs can provide all these functionalities simultaneously. Therefore, by utilizing this approach, one can easily print a multifunctional nanocomposite by using only the essential and minimal *two* components—ZnO QPIs and PEGDA—within an aqueous solution.

## Results and Discussion

### ZnO QPI Synthesis and Functionality in Photopolymerization

**Figure 1:**
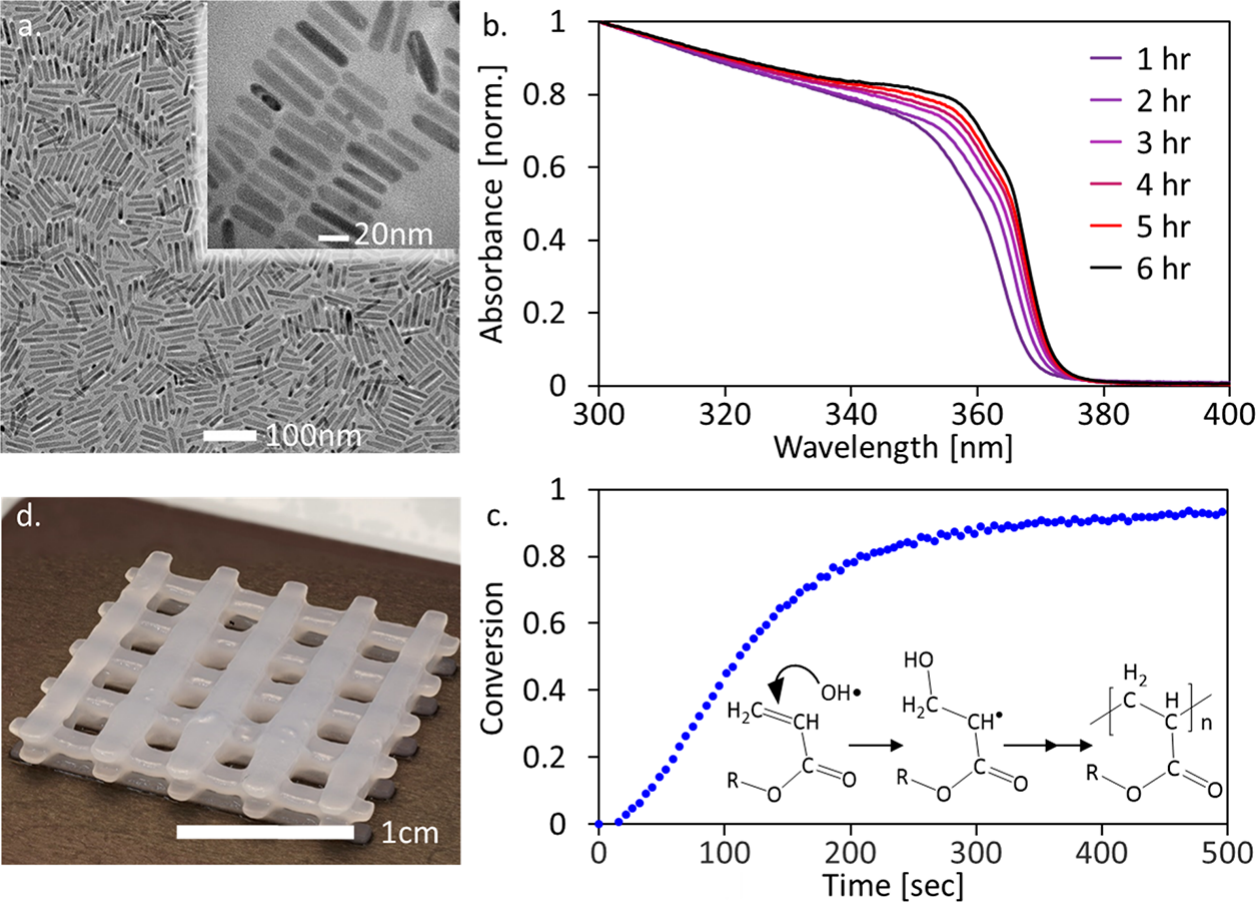
ZnO NRs as a QPI: (a) TEM image of the ZnO NRs. (b) Absorbance spectrum vs reaction time at 60 °C. (c) FTIR photopolymerization curves using ZnO QPIs showing the double-bond conversion ratio versus time for a water-based PEGDA hydrogel. Inset presents the QPI photoinitiation mechanism where hydroxyl radicals are formed photocatalytically upon irradiating the ZnO NRs and attack the C=C double bond in the acrylate group of the monomer. (d) QPI-based PEGDA hydrogel woodpile structure printed with a DLP 3D printer.

ZnO NRs were synthesized using Zn acetate dehydrate and KOH as zinc and oxygen precursors, respectively, and methanol as the solvent with a small amount of water. Previously reported syntheses were carried out in a two-step process. First, spherical ZnO nanocrystals were obtained in lower concentrations (below 0.1 M) of Zn(Ac)_2_. Then, the solution was concentrated (∼0.5 M), resulting in the formation of NRs, via oriented attachment of the spherical nanocrystals.^38^ Herein, we used the same mole ratio of KOH to Zn(Ac)_2_ (1.6:1) and combined the two consecutive steps by starting with a higher concentration of Zn(Ac)_2_ (∼0.3 M). Homogeneous rod-shaped nanocrystals were obtained, with a length of 35 ± 9 nm and a width of 8 ± 1 nm (Figure 1a and inset). The absorption onset of the final product ZnO NRs is at 370 nm (Figure 1b, black line).

The modified synthesis reduced the reaction time to a total of 6 h at 60 °C. Working with higher concentrations of precursors was previously found to accelerate the oriented attachment step.^39^ Applying this approach to both nucleation and directional growth stages enabled shortening the entire process while eliminating the evaporation step. These modifications are crucial for upscaling, which is essential for future applications in QPI-based nanocomposites.

The directional growth toward rod-shaped nanocrystals was characterized using TEM and UV–vis absorbance. Figure S1 shows the elongation of the nanocrystals during the first 5 h of the synthesis from 11 nm to 30 nm. The elongation and a slight thickening of the NRs is reflected in their UV–vis absorbance spectrum, noted by a red-shift of the peak from 350 nm to 357 nm for the final product (Figure 1b).

PEGDA was chosen as a water-based monomer to form the polymeric matrix, with the solution composition of choice containing a 1:1 PEGDA to water weight ratio. ZnO NRs in various amounts were mixed within the composition and acted as photoinitiators upon 365 nm irradiation.

To study the performances of the ZnO NPs as QPIs, their activity was examined by Fourier transform infrared (FTIR) spectroscopy, following the decrease of the monomer’s C=C double-bond feature at 1414 cm^–1^ upon curing.^40^ A 20 μL droplet of the formulation mixed with the QPIs was placed on the attenuated total reflection (ATR) crystal, followed by 35 mW cm^–2^ irradiation using a 365 nm LED source. Figure 1c presents traces of the C=C double-bond conversion kinetics, using 5 s scanning intervals, for PEGDA, after normalization of the absorption to an unchanged peak related to the C=O bond at 1720 cm^–1^.^41^ Nearly full conversion is seen (90% after 6 min). The inset shows the primary hypothesized photoinitiation mechanism that, according to our prior works, is based on facile photocatalytic formation of hydroxyl radicals upon irradiating the ZnO NRs in the aqueous solution.^22,24^

After we showed that the QPIs serve to create radicals for photopolymerization, we put them into action in DLP 3D printing instead of the commonly used organic photoinitiators. In addition to developing ink compositions to enrich the properties of the printed structures, another significant challenge in DLP is finding efficient water-soluble photoinitiators.^22,25^ Using DLP with a 385 nm projector as the light source, we fabricated woodpile structures by a printing process performed at 30 s irradiation for a single layer. The printed woodpile structure contains overhanging features and has precise and well-defined edges (Figure 1d). The ZnO NRs’ absorbance onset (∼370 nm) is shifted from the printer’s 385 nm nominal peak, and yet printing was possible; this can be explained due to the broad light spectrum of the DLP printer (Figure S3), combined with the red tail of the absorption of the NRs. This also attests to the photoinitiation efficiency of the ZnO-based QPIs, as rapid polymerization occurs despite their absorption spectrum being only in the tail of the DLP printer’s emission spectrum. We expect that using a designated DLP light source will outperform these results, enabling shorter exposure duration per layer or even decreasing the ZnO NRs’ concentration.

### QPI as a Mechanical Property Modifier

**Figure 2:**
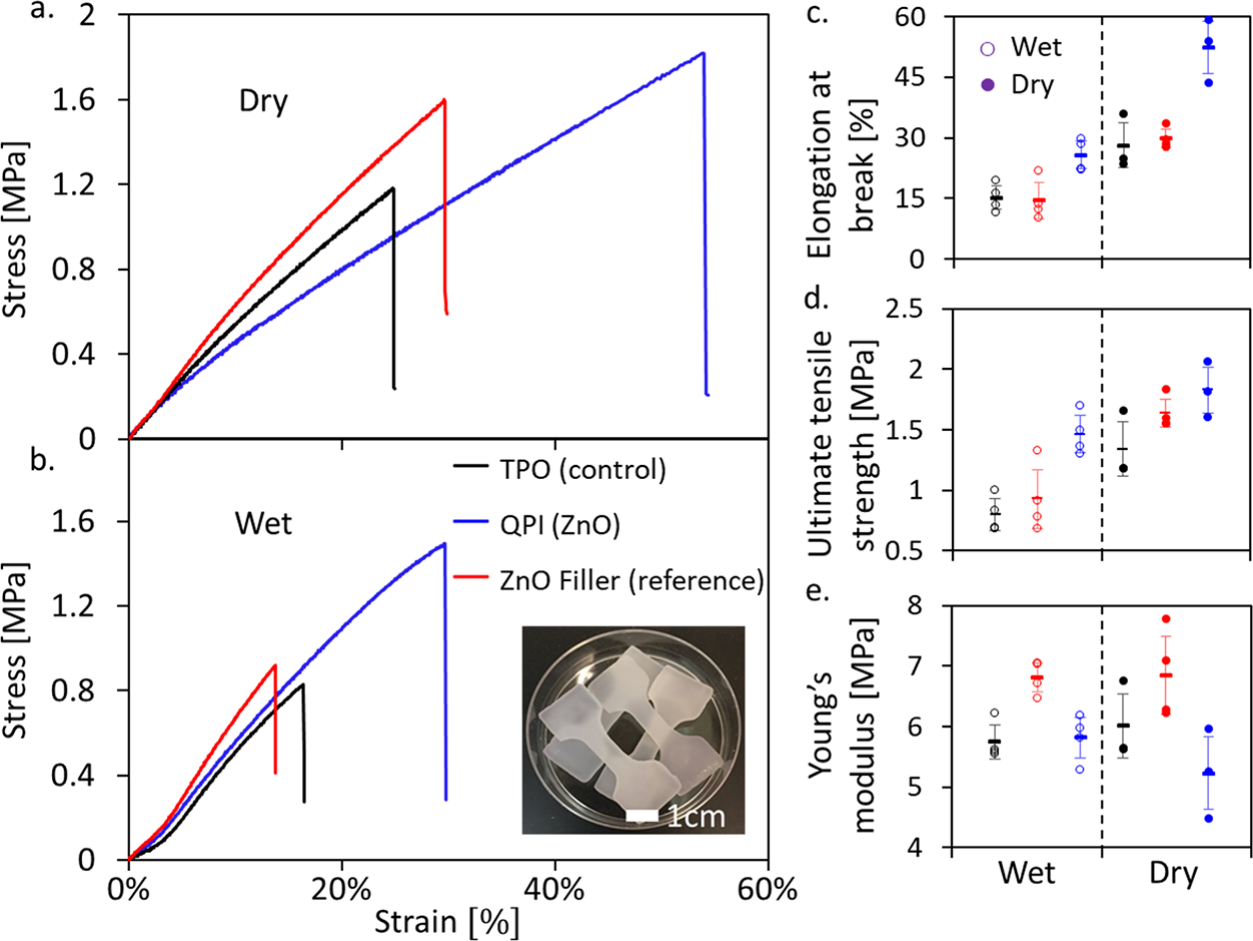
Stress–strain curves of PEGDA hydrogel dog-bone samples under (a) dry versus (b) wet conditions, comparing ZnO NRs acting as QPI (blue), reference system (red) with ZnO NRs only as a property modifier filler, and control system (black) photoinitiated by TPO. (c–e) Summary of the mechanical properties of PEGDA samples according to hydration conditions (unfilled and filled dots representing the wet and dry samples, respectively): (c) elongation at break, (d) ultimate tensile strength, (e) Young’s modulus. Horizontal lines are average values, and error bars are the standard deviation for each type of sample.

To evaluate the mechanical properties of the polymer nanocomposite material, dog-bone-shaped tensile test specimens were fabricated by molding (Figure 2b inset). The samples were then washed with 2-propanol (IPA) and placed in triple-distilled water (TDW) for 72 h. The water was replaced twice during that time.

The mechanical properties of PEGDA hydrogel samples were tested under both wet and dry conditions by using the dog-bone test samples. Wet conditions refer to samples taken directly from the aqueous solution for the measurements, while for dry conditions, the samples were allowed to dry out and kept in a desiccator for at least 72 h. In our study, after comparing the mechanical properties of samples with a different weight percentage of ZnO NRs, ranging between 0.5 and 1.5 wt % (Figure S4), we decided to continue with 1 wt % of ZnO NRs, which showed the highest elongation at break, tensile strength, and Young’s modulus. We compared QPI-driven PEGDA hydrogels containing 1 wt % of ZnO, a control system initiated by 0.1 wt % of TPO, and a ZnO filler reference sample with 1 wt % of ZnO NRs together with 0.1 wt % of TPO. Both reference and control samples were cured by exposure to a 405 nm LED light source to prevent any light-induced stimulation of the ZnO NRs. This approach allows us to differentiate selectively between the mechanical characteristics while using the ZnO NRs acting as both quantum photoinitiators and fillers or merely as fillers. In the reference sample in which both TPO and ZnO are present, ZnO NRs act merely as fillers, while TPO serves as the photoinitiator.

Figure 2a and b show stress–strain curves comparing the three sample types. Interestingly, significant differences are seen specifically for the sample with ZnO as the QPI. First, the results show a substantial increase in the film elongation at break (Figure 2c) for both the wet-swollen and the dry conditions, by 69% and 86%, respectively. Notably, when the ZnO NRs are used solely as fillers, they do not modify this characteristic compared with the TPO control sample. Correspondingly, the tensile strength (Figure 2d) under swollen conditions is significantly increased only for the ZnO QPI sample (by 83%). This trend is also seen in the dry samples, although moderated (an increase of 36% compared with the TPO control and just 12% compared to the ZnO filler reference). No significant difference in Young’s modulus is observed between the three samples in either wet or dry conditions (Figure 2e).

These results lead to the conclusion that organic photoinitiators and QPIs act differently. We hypothesize that while the former is consumed to form radicals homogeneously in the monomer solution, the latter creates areas with a higher concentration of continuously formed radicals around the SCNCs. This unique QPI behavior allows for a higher level of polymer branching in those areas. The further networking that is affiliated with the initiation mechanism of ZnO QPIs does not change the Young’s modulus but can increase the ultimate tensile strength and elongation before failure. In the case of using ZnO NRs only as fillers with TPO as the photoinitiator, neither the complexity of branching nor the mechanical properties of the final product change with the addition of the fillers

### Post-polymerization Photocatalytic Activity

**Figure 3:**
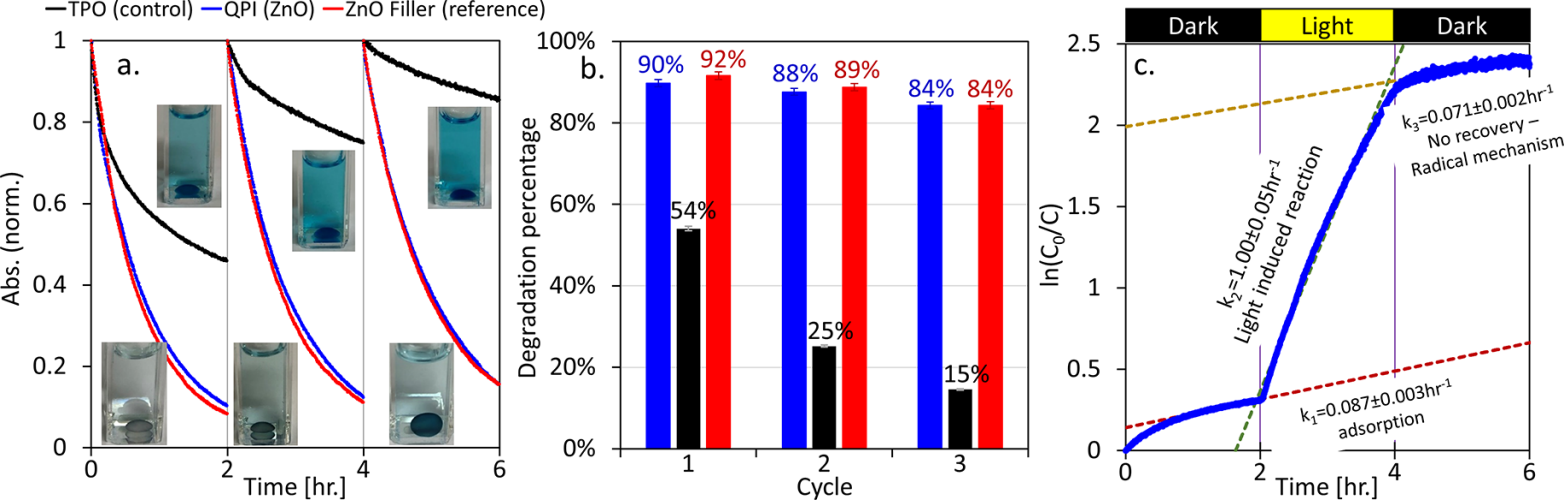
(a) Degradation of MB in water by ZnO-containing PEGDA discs. (a) The temporal dependence of the absorption of the dye main peak at 655 nm during three cycles of exposure to a 365 nm LED source; comparison between PEGDA films containing 1 wt % of ZnO NRs which act as QPI (blue), as a property modifier filler (red), and a control system (black) photoinitiated by TPO. (b) The percentage of degradation at the end of each cycle, calculated as the relative difference in absorbance between the initial and final stage. (c) Demonstration of the photocatalytic mechanism. Shown is the relative MB concentration in log scale versus time starting in the dark for 2 h, followed by 2 h under 365 nm irradiation, and ending again with 2 h in the dark.

In contrast to organic photoinitiators that react stoichiometrically and are consumed during the polymerization reaction, QPIs create radicals in a catalytic manner and can maintain their characteristics even when embedded in the nanocomposite polymer, endowing the system with photocatalytic activity. This unique behavior is clearly demonstrated by a multicycle dye degradation test, in which polymerized molded discs of PEGDA were placed in solutions with methylene blue (MB) and irradiated at 365 nm by an LED (Figure 3a). The photodegradation was evaluated for three cycles of addition of fresh MB. In the first cycle, the control sample showed degradation of MB by ∼50% after 2 h, and this value diminished significantly to 25% and 15% in the second and third cycles, respectively. In contrast, the ZnO-containing samples showed a considerably higher degradation of ∼90% in the same 2 h period, with just a small decrease in the second and third cycles (Figure 3b). The dye degradation percentage of MB for the ZnO-containing samples was nearly six times higher than the control reference sample polymerized using TPO, after the three consecutive cycles.

As to the origin of the decrease in the dye concentration in solution, the first mechanism, irrespective of the ZnO photocatalytic activity, is related to the function of the hydrogel discs as MB adsorbents. This mechanism is the source of the decrease in the MB solution content for the control discs. The discs are indeed changing in color and appear dark blue due to the presence of adsorbed MB. Moreover, a saturation of the adsorbent power is already significant after just one cycle. In contrast, the ZnO-containing discs show a persistent degradation capacity over several cycles of dye additions. This is attributed to the photocatalytic mechanism induced by the ZnO NRs within the discs. To more clearly distinguish the dark activity from the photocatalytic activity, the ZnO QPI sample was introduced to the dye solution without exposure to light for 2 h, followed by 2 h of direct illumination, and then 2 h in the dark again, to allow also for possible dye recovery (Figure 3c). In the dark, only some decrease in MB concentration is seen and then is saturated due to the diminished adsorbent power of the PEGDA film. Turning on the illumination leads to a significantly hastened decrease in the MB concentration, which follows first-order kinetics. Upon turning the illumination off, the MB concentration decrease subsides. Furthermore, there is no recovery of the MB, indicating a photoinduced and irreversible reaction. The saturated rate of dark activity (*k*_1_, red dashed line), calculated by linear fitting of the curve after 1 h of initial adsorption, nearly matches the rate in the second dark period. We observe more than 10 times faster degradation of the dye upon illumination compared to the dark conditions, and no recovery is seen. The ZnO NRs may directly reduce MB to leucomethylene blue, which is easily reversible back to MB in the presence of oxygen. The irreversibility of the dye upon stopping illumination suggests that the ZnO NRs within the discs degrade MB beyond a mere reduction. This characteristic performance allows ZnO QPI-driven films and 3D objects to be used for purification by photodegradation processes of organic pollutants.

**Figure 4:**
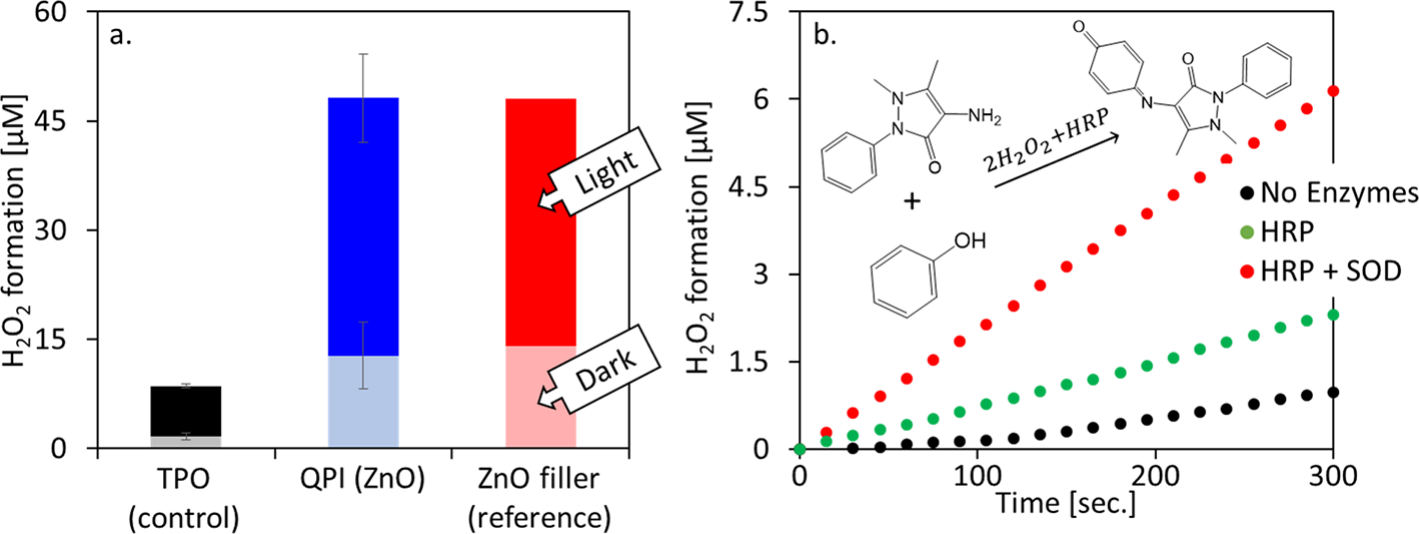
(a) Hydrogen peroxide formation measured for PEGDA films in the dark and under illumination of a 365 nm LED source for 2 h. The amount of hydrogen peroxide was detected using an enzymatic assay. (b) Initial rates of reactive oxygen species production for PEGDA films under illumination.

To further investigate the photocatalytic mechanism of action of the nanocomposites with embedded ZnO NRs, we utilized an enzymatic assay to identify the formed radical species. Photocatalytic formation of hydrogen peroxide (H_2_O_2_) was measured via a colorimetric absorption assay, measuring the production over time of quinoneimine in the presence of horseradish peroxidase (HRP).^42^ Adding the superoxide dismutase (SOD) enzyme enables measurement of the formation of superoxide (^•^O_2_^–^) as well, from the difference in H_2_O_2_ formation with HRP and SOD together versus HRP alone. After 2 h, in the dark activity test, ZnO-containing films manifest eight times higher H_2_O_2_ formation when compared to the control system (Figure 4a). This might be attributed to the presence of free catalytic Zn^2+^ ions in the solution and oxygen vacancies in the nanocrystal,^43^ also referred to as dangling Zn sites, which are highly abundant on the Zn-rich facet of the ZnO NRs. Interestingly, under illumination, the results show a 5-fold increase in photoinduced formation of H_2_O_2_ molecules for the ZnO-containing films, both as QPI and as filler, over the TPO-initiated control system (Figure 4a). Figure 4b shows the first 5 min of quinoneimine formation with and without SOD for the QPI films. While the initial rate for HRP alone is 0.27 μM min^–1^, adding SOD increases it more than 3-fold to 1.03 μM min^–1^. With HRP alone, H_2_O_2_ may form via a direct two-electron reduction process of oxygen. By combining SOD with HRP testing, an additional route is made possible through one-electron reduction to form superoxide radicals that subsequently react in the presence of SOD to form H_2_O_2_. The terephthalic acid (TPA) assay for hydroxyl radical detection showed no significant results. While hydroxyl radicals play an important part in the polymerization process itself,^17,24^ the main post-polymerization photocatalytic activity of the nanocomposite is attributed to the formation of superoxide and hydrogen peroxide, which are characterized by a longer lifetime and higher stability and selectivity than hydroxyl radicals.^44,45^ These characteristics are highly relevant for the post-polymerization photocatalytic activity, since the active sites are embedded within the polymeric matrix and the radicals should travel through it at a low diffusion rate into the surrounding medium.

### Antimicrobial Activity of ZnO-Induced Nanocomposite Polymers

**Figure 5:**
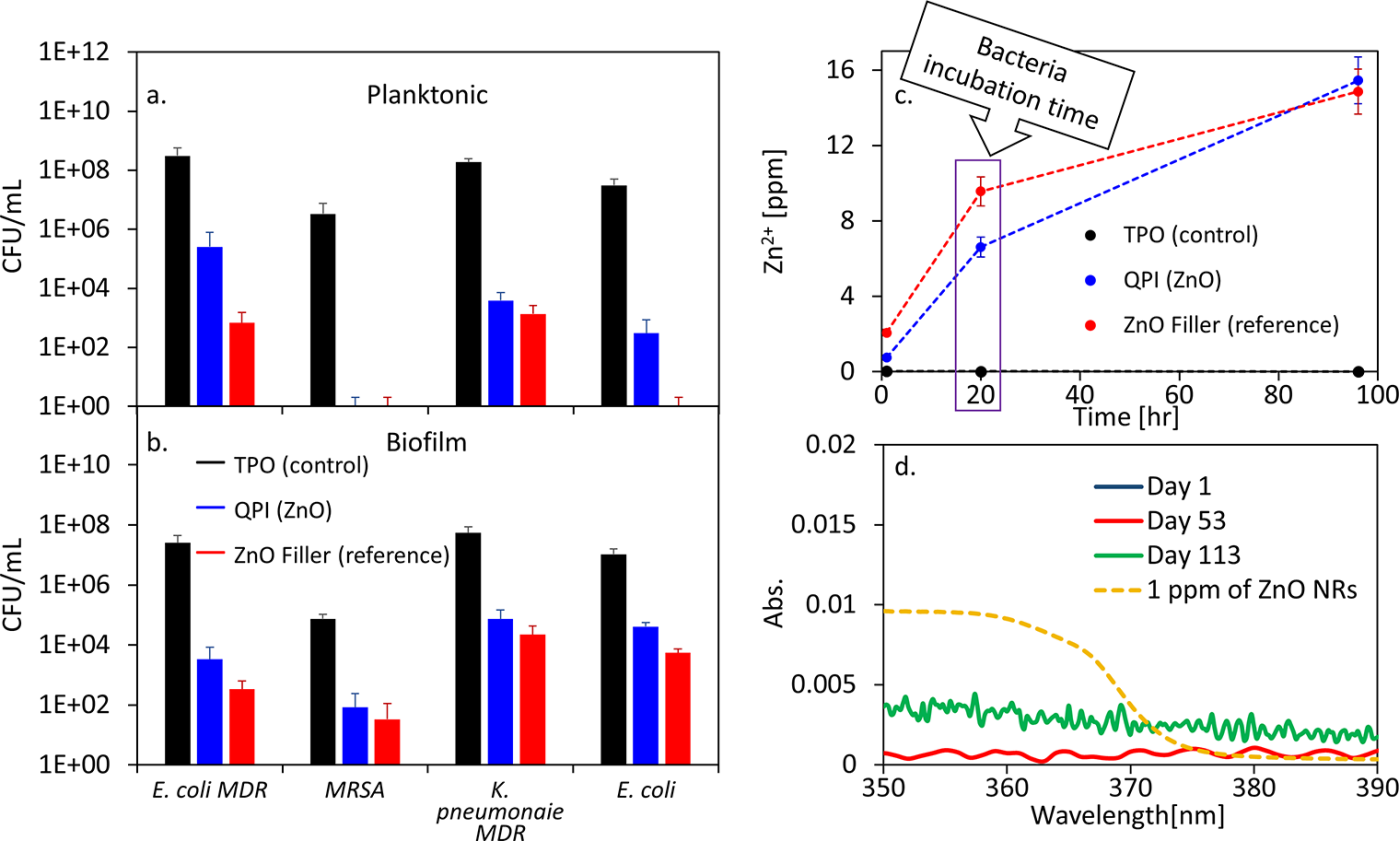
Antibacterial activity test results of (a) planktonic and (b) biofilm bacteria. The number of viable bacteria is presented as colony-forming units (CFUs) per mL. Migration of Zn^2+^ ions (c) and ZnO NRs (d) calculated by ICP-MS and UV–vis absorbance, respectively. ZnO NR migration was measured only for the QPI films and compared to a 1 ppm ZnO NR solution in water (yellow dashed line).

An additional unique aspect of QPI-based nanocomposites is their potential for post-polymerization antimicrobial functionality. To test the antimicrobial activity of the ZnO-embedded PEGDA nanocomposite, a set of samples of PEGDA hydrogels was cured in a mold to produce small round surfaces (discs) that can fit into a 24-well plate, for the cultivation of various multi-drug-resistant bacteria and a virus. Antimicrobial efficacy was tested against both planktonic (medium-suspended) and surface-attached (biofilm) bacteria, as presented in Figure 5a and b, respectively. We note that the light-induced antimicrobial activity was not tested herein, due to complications inherent in utilizing UV illumination, antimicrobial in its own right, for such a study. Compared to the TPO-initiated control films, the ZnO-containing surfaces manifest significant antibacterial activity. This is the case for both the ZnO NRs as QPI or just as filler. For *E. coli*, the planktonic measurement shows nearly complete bacteria eradication, while the antibiofilm activity demonstrated a more than 3-log reduction in bacteria concentration compared to the control. This strong activity is also observed for various multi-drug-resistant strains, including *E. coli* MDR, MRSA, and *K. pneumoniae* MDR.

**Figure 6:**
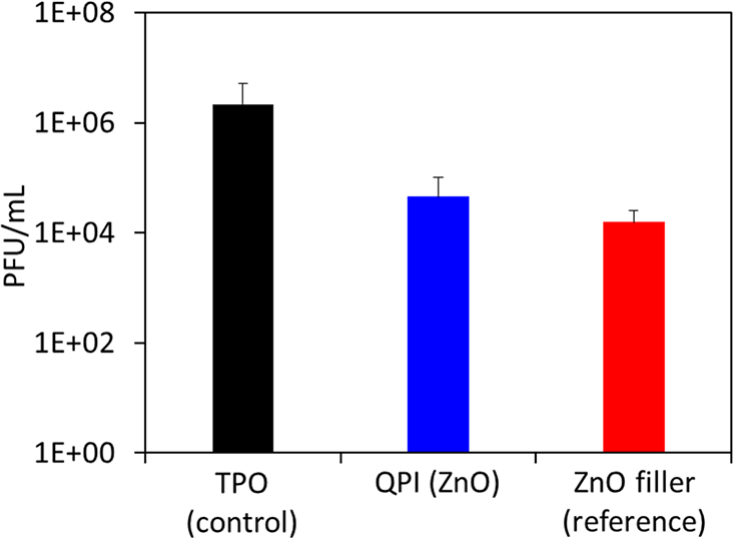
Antiviral activity test results of the MS2 phage. The number of viable phages is presented as plaque-forming units (PFUs) per mL.

Antiviral activity of the ZnO-induced nanocomposites was also assessed, based on an MS2 phage assay. Significant antiviral activity exhibited by an ∼2-log reduction in phage titer was shown for the samples containing ZnO (Figure 6).

To address the antimicrobial mechanism, we studied the leaching of ZnO NRs and Zn^2+^ ions from the nanocomposite over time, as these are known to induce antimicrobial activity (Figure 5c). Film samples containing ZnO NRs that underwent the same treatment as the antimicrobial surfaces, namely, storage in TDW for 72 h followed by extensive washing to remove loosely hanging residues, were then placed in an aqueous solution. First, UV–vis spectrophotometric measurements did not show any signature of migration of ZnO NRs into solution (Figure 5d). This is consistent with their large size and embedding in the hydrogel network. Second, ICP-MS analysis was also performed at different soaking times in water to measure the leaching of Zn^2+^ ions. A gradual increase in Zn^2+^ ions was observed over time. At 20 h, the incubation time for the antibacterial studies, more than 6 ppm of Zn^2+^ ions were measured in solution, for both the QPI and filler systems (Figure 5c, blue and red lines, respectively). Such a release of the Zn^2+^ ions can contribute to the antibacterial functionality of the nanocomposites.

## Conclusion

In this research, we demonstrated that QPIs can serve as an all-in-one approach for making functional nanocomposites by acting as both photocatalytic initiators and property modifiers in a polymer. We showed a ZnO NR-based model system for multifunctional QPIs that was able to increase the tensile strength and elongation at break of PEGDA hydrogels by up to 85%, unlike the use of the same ZnO NRs as fillers. This is attributed to the unique photocatalytic photoinitiation action of the ZnO QPIs, which creates a stronger cross-linked networked composite. At the same time, the ZnO QPIs provided the resulting PEGDA hydrogels with photocatalytic and antimicrobial activities. These combined activities make them useful for implementation in biomedical photopolymers, eliminating the need for potentially harmful common organic photoinitiators and the need for additional reinforcement fillers. A simple formulation with merely ZnO NRs and PEGDA in aqueous solution addresses all of these needs, significantly simplifying the system and reducing barriers to its regulatory acceptance in future bio- and medical application scenarios. Moreover, we showed two fabrication showcase methods: traditional molding and 3D DLP printing, a stereolithography additive manufacturing process that can obtain hydrogels with complex architectures. This creates numerous flexible opportunities to design the structures of the desired PEGDA hydrogels for tailored functionality. These multifunctional abilities demonstrate the potential benefits of this approach, especially when it acts as a multipurpose ink in additive manufacturing systems. With improved understanding and control of the QPI curing mechanism, we expect that such systems can be utilized as multifunctional photoinitiators in applications ranging from 3D bioprinting to advanced active filtering systems.

## Methods

### Chemicals

Zinc acetate dehydrate (≥98%), potassium hydroxide (KOH, 90%), diphenyl (2,4,6-trimethylbenzoyl)phosphine oxide (TPO, 97%), phosphate buffer saline (PBS, tablet), phenol (≥99.5%), hydrogen peroxide (30%), methylene blue (MB), oleylamine (98%), octanethiol (≥98.5%), and superoxide dismutase (SOD) were purchased from Sigma-Aldrich. Polyethylene glycol (600) diacrylate (PEGDA, SR-610) was purchased from Sartomer-Arkema. Methanol and IPA were purchased from a local warehouse. Horseradish peroxidase (HRP) was purchased from Thermo Scientific. 4-Aminoantipyrine (≥98.0%) was purchased from Fluka Analytical. All chemicals were used as received without further purification.

### ZnO NR Synthesis

The procedure is a modification of a previously reported synthesis.^46^ We perform a one-step colloidal synthesis of ZnO NRs on a large scale of about 9 g per batch, by dissolving 29.4 g (0.5 M) of Zn(Ac)_2_·2H_2_O in water (6 mL) and methanol (315 mL), heating to a reflux, then adding 14.58 g (1.5 M) of KOH in methanol (172.5 mL), and holding under Ar flow for 6 h. The synthesis yields ligand-free ZnO NRs dispersible in polar solvents.

### ZnO NRs’ Structural Characterization

For improved structural characterization, the obtained nanocrystals underwent a ligand exchange procedure with octanethiol and oleylamine, rendering them soluble in toluene. The sample was cleaned from excess organic ligands using precipitation with alcohol as an antisolvent and then deposited on transmission electron microscope (TEM) grids from the toluene solution. The characterization was then performed using a Tecnai T12 G2 Spirit TEM. Absorption spectra were taken using a JASCO V-570 spectrophotometer.

### QPI-Induced Nanocomposite Polymers

To examine the ability of QPI to modify the mechanical properties of polymers, a model system was studied. This system is based on ZnO NRs as fillers and as photoinitiators for the curing of biocompatible PEGDA photopolymers in a mold, forming a cross-linked hydrogel. To follow the photopolymer curing process, ATR-FTIR measurements were conducted using a Thermo Scientific Nicolet iS50 instrument and a diamond ATR crystal. After curing, to avoid the migration of unreacted photoactive species from the polymer surface, all PEGDA nanocomposite samples were washed once with IPA and then kept in TDW for 72 h, during which the water was replaced twice.

### DLP Printing

The printing composition for digital light processing printing was the same as for the mold compositions, with a 1:1 PEGDA to water weight ratio and 1 wt % of ZnO NRs. To fabricate 3D objects, predesigned STL models were 3D printed using a DLP 3D printer (Asiga MAX X35, Australia) equipped with a 385 nm light source. Printing was performed using a light intensity of 15 mW cm^–2^. STL models were sliced to one layer of “burn-in” with 0.1 mm thickness that was exposed for 50 s. The thickness of all other layers was set to 0.2 mm with a 30 s exposure for each layer.

### Tensile Test

An INSTRON tensile machine was used to evaluate the mechanical parameters of the polymers in a dog-bone shape (dimensions in Figure S5). The rate of extension was set to 5 mm min^–1^. For each type of sample, at least three repetitions were carried out. The test results were then analyzed to create a stress–strain curve from which Young’s modulus, maximum tensile strength, and elongation at break values were taken. For the wet (swollen) condition, the test was carried out right after taking the specimen out of the water, while the dry samples were dried and placed in a desiccator for 72 h before testing.

### Methylene Blue Dye Degradation

PEGDA hydrogel nanocomposite films, containing 1 wt % of ZnO NRs, were placed in a cuvette filled with MB that was dispersed in an aqueous solution (∼1 OD @ 655 nm) and was irradiated at 365 nm with 35 mW cm^–2^ intensity for 2 h. After the first degradation cycle, the dye concentration was renewed to the initial value and two more identical cycles were carried out. The degradation of MB was measured by following the decrease in the dye absorption peak, using an Ocean Optic spectrophotometer, in 10 s scanning intervals.

### Photocatalytic Formation of Reactive Oxygen Species

To determine the formation of hydrogen peroxide and superoxide, enzymatic assays were used based on a published method.^42^ An enzymatic solution was prepared by mixing together 4-aminoantipyrine (200 μL, 8.125 mg mL^–1^), phenol (600 μL, 79 mg mL^–1^), PBS (200 μL, 0.1 M), and HRP (20 μL (0.4 mg mL^–1^) in TDW (1 mL). The PEGDA hydrogel nanocomposite films were inserted into the enzymatic solution and irradiated. In parallel, the dark activity was assessed by covering the samples with aluminum foil. The absorption of 1 mL sampled from the solution was measured after 2 h, and the concentration of the produced quinoneimine dye was calculated using its molar extinction coefficient at 500 nm (12 mM^–1^ cm^–1^). The concentration of the formed H_2_O_2_ was calculated by subtracting the absorption of the enzyme-free samples from the measured absorption and multiplying it by two, as two H_2_O_2_ molecules are required to form one quinoneimine molecule upon reaction with HRP. To measure the contribution of superoxide, SOD (25 μL, 1 mg mL^–1^) was also mixed in the solution, which enhances the formation of quinoneimine by converting superoxide into oxygen and H_2_O_2_.

### Antibacterial and Antiviral Activity

For the antibacterial activity studies, *Escherichia coli* ATCC 25922, MDR *E. coli* ATCC BAA-2452, MDR *Klebsiella pneumonia* ATCC BAA-1898, and methicillin-resistant *Staphylococcus aureus* (MRSA) ATCC 43300 were grown overnight in Mueller-Hinton (MH) broth at 37 °C (250 rpm). The overnight cultures were diluted in 1% MH medium to a desired initial OD of 0.01 for MRSA and 0.3 for *E. coli* and *K. pneumoniae*. For MRSA, the medium was supplemented with 0.2% glucose. In addition, for MRSA, the disc-shaped samples of the PEGDA hydrogel nanocomposite were gently rinsed in sterile DDW for 15 min and then glued to the bottom of each well in a 24-well plate. Then, a diluted culture (1 mL) was added on top of the discs. For *E. coli* and *K. pneumoniae*, the wells were first filled with diluted culture (1 mL), and the discs were placed on top of the liquid phase. The plates were incubated overnight at 37 °C. The following day, samples from the liquid phase of each well were taken for a viable count of the planktonic bacteria. To eliminate free bacteria, the discs were taken out and rinsed three times in sterile DDW. The biofilm formed on each disc was scraped off and suspended in fresh media, from which samples were taken for serial dilution and a viable count.

For antiviral activity studies, MS2 phage titer was determined and diluted to a 10^7^ PFU mL^–1^ suspension. A 1 mL amount of diluted phage suspension was transferred to each well of a 24-well plate, and disc-shaped samples of the PEGDA hydrogel nanocomposite were placed on top of the liquid. The plates were then incubated at 37 °C for approximately 18 h. At the same time, the host bacteria (*E. coli* C-3000 ATCC 155970) were incubated in Luria–Bertani (LB) medium at 37 °C (250 rpm) overnight. The following day, the culture of the host bacteria was refreshed in the same medium and incubated for 2 h until reaching mid log phase. The refreshed host (100 μL) was added to soft agar (5 mL) prewarmed to 50 °C and immediately poured onto an LB-agar plate. After the plates were set, the phage suspension of each well was sampled, serially diluted, and drop-plated onto the premade soft agar plate. The plate was incubated at 37 °C overnight, after which, plaque-forming units were calculated.

Statistical analysis for all antimicrobial experiments is based on three biological repeats with two internal technical repeats.

## Supporting Information Available

ZnO NR synthesis process, hydrogel swelling effect, optical characterization of ZnO and DLP projector, mechanical properties of nanocomposites with different amounts of ZnO, dimensions of the tensile test dog-bone specimen (PDF)

## References

[ref1] Li Q.; Kulikovski J.; Doan D.; Tertuliano O. A.; Zeman C. J.; Wang M. M.; Schatz G. C.; Gu X. W. Mechanical Nanolattices Printed Using Nanocluster-Based Photoresists. Science 2022, 378, 768–773. 10.1126/science.abo6997. 36395243

[ref2] Fu S.; Sun Z.; Huang P.; Li Y.; Hu N. Some Basic Aspects of Polymer Nanocomposites: A Critical Review. Nano Mater. Sci. 2019, 1, 2–30. 10.1016/j.nanoms.2019.02.006.

[ref3] Jordan J.; Jacob K. I.; Tannenbaum R.; Sharaf M. A.; Jasiuk I. Experimental Trends in Polymer Nanocomposites – a Review. Mater. Sci. Eng., A 2005, 393, 1–11. 10.1016/j.msea.2004.09.044.

[ref4] Gigi S.; Naor T.; Waiskopf N.; Stone D.; Natan M.; Jacobi G.; Levi A.; Remennik S.; Levi-Kalisman Y.; Banin E.; Banin U. Photoactive Antimicrobial CuZnO Nanocrystals. J. Phys. Chem. C 2022, 126, 18683–18691. 10.1021/acs.jpcc.2c05109.

[ref5] Zhang D.; Yang J.; Bao S.; Wu Q.; Wang Q. Semiconductor Nanoparticle-based Hydrogels Prepared *via* Self-Initiated Polymerization under Sunlight, Even Visible Light. Sci. Rep. 2013, 3, 1399 10.1038/srep01399. 23466566 PMC3590559

[ref6] Liang X. Visualization of Nanomechanical Properties of Polymer Composites Using Atomic Force Microscopy. Polym. J. 2023, 55, 913 10.1038/s41428-023-00790-9.

[ref7] Li H.; Ai D.; Ren L.; Yao B.; Han Z.; Shen Z.; Wang J.; Chen L.-Q.; Wang Q. Scalable Polymer Nanocomposites with Record High-Temperature Capacitive Performance Enabled by Rationally Designed Nanostructured Inorganic Fillers. Adv. Mater. 2019, 31, 1900875 10.1002/adma.201900875. 30977229

[ref8] Panfil Y. E.; Oded M.; Banin U. Colloidal Quantum Nanostructures: Emerging Materials for Display Technologies. Angew. Chem., Int. Ed. 2018, 57, 4274–4295. 10.1002/anie.201708510. PMC6001641 28975692

[ref9] Waiskopf N.; Banin U. Colloidal Quantum Materials for Photocatalytic Applications. In Curious2018; Betz U., Ed.; Springer Chem., 2019; pp 565–566, 10.1007/978-3-030-16061-6_12.

[ref10] Srivastava S.; Schaefer J. L.; Yang Z.; Tu Z.; Archer L. A. 25th Anniversary Article: Polymer–Particle Composites: Phase Stability and Applications in Electrochemical Energy Storage. Adv. Mater. 2014, 26, 201–234. 10.1002/adma.201303070. 24323839

[ref11] Parameswaranpillai J.; Das P.; Ganguly S. Quantum Dots and Polymer Nanocomposites: Synthesis, Chemistry, and Applications; CRC Press, 2023.

[ref12] Won Y. H.; Cho O.; Kim T.; Chung D. Y.; Kim T.; Chang H.; Jang H.; Lee J.; Kim D.; Jang E. Highly Efficient and Stable InP/ZnSe/ZnS Quantum Dot Light-emitting Diodes. Nature 2019, 575, 634–638. 10.1038/s41586-019-1771-5. 31776489

[ref13] Wegner K. D.; Hildebrandt N. Quantum Dots: Bright and Versatile in vitro and *in vivo* Fluorescence Imaging Biosensors. Chem. Soc. Rev. 2015, 44, 4792–4834. 10.1039/C4CS00532E. 25777768

[ref14] Xiong H.-M.; Wang Z.-D.; Liu D.-P.; Chen J.-S.; Wang Y.-G.; Xia Y.-Y. Bonding Polyether onto ZnO Nanoparticles: An Effective Method for Preparing Polymer Nanocomposites with Tunable Luminescence and Stable Conductivity. Adv. Funct. Mater. 2005, 15, 1751–1756. 10.1002/adfm.200500167.

[ref15] Beek W. J. E.; Wienk M. M.; Janssen R. A. J. Efficient Hybrid Solar Cells from Zinc Oxide Nanoparticles and a Conjugated Polymer. Adv. Mater. 2004, 16, 1009–1013. 10.1002/adma.200306659.

[ref16] Ates B.; Koytepe S.; Ulu A.; Gurses C.; Thakur V. K. Chemistry, Structures, and Advanced Applications of Nanocomposites from Biorenewable Resources. Chem. Rev. 2020, 120, 9304–9362. 10.1021/acs.chemrev.9b00553. 32786427

[ref17] Waiskopf N.; Magdassi S.; Banin U. Quantum Photoinitiators: Toward Emerging Photocuring Applications. J. Am. Chem. Soc. 2021, 143, 577–587. 10.1021/jacs.0c10554. 33353293

[ref18] Kuriacose J. C.; Markham M. C. Mechanism of the Photo-Initiated Polymerization of Methyl Methacrylate at Zinc Oxide Surfaces. J. Phys. Chem. 1961, 65, 2232–2236. 10.1021/j100829a032.

[ref19] Hoffman A. J.; Yee H.; Mills G.; Hoffmann M. R. Photoinitiated Polymerization of Methyl Methacrylate Using Q-Sized Zinc Oxide Colloids. J. Phys. Chem. 1992, 96, 5540–5546. 10.1021/j100192a066.

[ref20] Hoffman A. J.; Yee H.; Mills G.; Hoffmann M. R. Q-sized Cadmium Sulfide: Synthesis, Characterization, and Efficiency of Photoinitiation of Polymerization of Several Vinylic Monomers. J. Phys. Chem. 1992, 96, 5546–5552. 10.1021/j100192a067.

[ref21] Verbitsky L.; Waiskopf N.; Magdassi S.; Banin U. A Clear Solution: Semiconductor Nanocrystals as Photoinitiators in Solvent Free Polymerization,. Nanoscale 2019, 11, 11209–11216. 10.1039/C9NR03086G. 31157812

[ref22] Pawar A. A.; Halivni S.; Waiskopf N.; Ben-Shahar Y.; Soreni-Harari M.; Bergbreiter S.; Banin U.; Magdassi S. Rapid Three-Dimensional Printing in Water Using Semiconductor–Metal Hybrid Nanoparticles as Photoinitiators. Nano Lett. 2017, 17, 4497–4501. 10.1021/acs.nanolett.7b01870. 28617606

[ref23] Shukla S.; Pandey P. C.; Narayan R. J. Tunable Quantum Photoinitiators for Radical Photopolymerization. Polymers 2021, 13, 2694 10.3390/polym13162694. 34451234 PMC8398557

[ref24] Pinkas A.; Waiskopf N.; Gigi S.; Naor T.; Layani A.; Banin U. Morphology Effect on Zinc Oxide Quantum Photoinitiators for Radical Polymerization. Nanoscale 2021, 13, 7152–7160. 10.1039/D1NR00896J. 33889919

[ref25] Pawar A. A.; Saada G.; Cooperstein I.; Larush L.; Jackman J. A.; Tabaei S. R.; Cho N.; Magdassi S. High-Performance 3D Printing of Hydrogels by Water-Dispersible Photoinitiator Nanoparticles. Sci. Adv. 2016, 2, 10.1126/sciadv.1501381. PMC4820376 27051877

[ref26] Chen M.; Zhong M.; Johnson J. A. Light-Controlled Radical Polymerization: Mechanisms, Methods, and Applications. Chem. Rev. 2016, 116, 10167–10211. 10.1021/acs.chemrev.5b00671. 26978484

[ref27] Ruhland K.; Habibollahi F.; Horny R. Quantification and Elucidation of the UV-Light Triggered Initiation Kinetics of TPO and BAPO in Liquid Acrylate Monomer. J. Appl. Polym. Sci. 2020, 137, 48357 10.1002/app.48357.

[ref28] Lu H.; Liu Y.; Guo J.; Wu H.; Wang J.; Wu G. Biomaterials with Antibacterial and Osteoinductive Properties to Repair Infected Bone Defects. Int. J. Mol. Sci. 2016, 17, 334 10.3390/ijms17030334. 26950123 PMC4813196

[ref29] Mijnendonckx K.; Leys N.; Mahillon J.; Silver S.; Van Houdt R. Antimicrobial Silver: Uses, Toxicity and Potential for Resistance. BioMetals 2013, 26, 609–21. 10.1007/s10534-013-9645-z. 23771576

[ref30] Raghunath A.; Perumal E. Metal Oxide Nanoparticles as Antimicrobial Agents: A Promise for the Future. Int. J. Antimicrob. Agents 2017, 49, 137–152. 10.1016/j.ijantimicag.2016.11.011. 28089172

[ref31] Kumar R.; Umar A.; Kumar G.; Nalwa H. S. Antimicrobial Properties of ZnO Nanomaterials: A Review. Ceram. Int. 2017, 43, 3940–3961. 10.1016/j.ceramint.2016.12.062.

[ref32] He W.; Kim H.; Wamer W. G.; Melka D.; Callahan J. H.; Yin J. Photogenerated Charge Carriers and Reactive Oxygen Species in ZnO/Au Hybrid Nanostructures with Enhanced Photocatalytic and Antibacterial Activity. J. Am. Chem. Soc. 2014, 136, 750–757. 10.1021/ja410800y. 24354568

[ref33] Chong W. J.; Shen S.; Li Y.; Trinchi A.; Pejak D.; Kyratzis I. L.; Sola A.; Wen C. Additive Manufacturing of Antibacterial PLA-ZnO Nanocomposites: Benefits, Limitations and Open Challenge. J. Mater. Sci. Technol. 2022, 111, 120–151. 10.1016/j.jmst.2021.09.039.

[ref34] Yamamoto O. Influence of Particle Size on the Antibacterial Activity of Zinc Oxide. Int. J. Inorg. Mater. 2001, 3, 643–646. 10.1016/S1466-6049(01)00197-0.

[ref35] Embden J. V.; Gross S.; Kittilstved K. R.; Gaspera E. D. Colloidal Approaches to Zinc Oxide Nanocrystals. Chem. Rev. 2023, 123, 271–326. 10.1021/acs.chemrev.2c00456. 36563316

[ref36] Takatsuka T.; Tanaka K.; Iijima Y. Inhibition of Dentine Demineralization by Zinc Oxide: *In vitro* and *in situ* Studies. Dent. Mater. J. 2005, 21, 1170–7. 10.1016/j.dental.2005.02.006. 16046230

[ref37] Samanta D.; Jena P. Zn in the + III Oxidation State. J. Am. Chem. Soc. 2012, 134, 8400–8403. 10.1021/ja3029119. 22559713

[ref38] Pacholski C.; Kornowski A.; Weller H. Self-Assembly of ZnO: From Nanodots to Nanorods. Angew. Chem., Int. Ed. 2002, 41, 1188–1191. 10.1002/1521-3773(20020402)41:7<1188::AID-ANIE1188>3.0.CO;2-5. 12491255

[ref39] Sun B.; Sirringhaus H. Solution-Processed Zinc Oxide Field-Effect Transistors Based on Self-Assembly of Colloidal Nanorods. Nano Lett. 2005, 5, 2408–2413. 10.1021/nl051586w. 16351187

[ref40] Visentin A. F.; Dong T.; Poli J.; Panzer M. J. Rapid, Microwave-Assisted Thermal Polymerization of Poly (ethylene glycol) Diacrylate-Supported Ionogels. J. Mater. Chem. A 2014, 2, 7723–7726. 10.1039/c4ta00907j.

[ref41] Magalhães L. S. S. M.; Andrade D. B.; Bezerra R. D. S.; Morais A. I. S.; Oliveira F. C.; Rizzo M. S.; Silva-filho E. C.; Lobo A. O. Nanocomposite Hydrogel Produced from PEGDA and Laponite for Bone Regeneration. J. Funct. Biomater. 2022, 13, 53 10.3390/jfb13020053. 35645261 PMC9149996

[ref42] Stone D.; Ben-Shahar Y.; Waiskopf N.; Banin U. The Metal Type Governs Photocatalytic Reactive Oxygen Species Formation by Semiconductor-Metal Hybrid Nanoparticles. ChemCatChem. 2018, 10, 5119–5123. 10.1002/cctc.201801306.

[ref43] Xu X.; Chen D.; Yi Z.; Jiang M.; Wang L.; Zhou Z.; Fan X.; Wang Y.; Hui D. Antimicrobial Mechanism Based on H_2_O_2_ Generation at Oxygen Vacancies in ZnO Crystals. Langmuir 2013, 29, 5573–5580. 10.1021/la400378t. 23570415

[ref44] Kulkarni A. C.; Kuppusamy P.; Parinandi N. Oxygen, the Lead Actor in the Pathophysiologic Drama: Enactment of the Trinity of Normoxia, Hypoxia, and Hyperoxia in Disease and Therapy. ARS 2007, 9, 1717–1730. 10.1089/ars.2007.1724. 17822371

[ref45] Diaz J. M.; Plummer S. Production of extracellular reactive oxygen species by phytoplankton: past and future directions. J. Plankton Res. 2018, 40, 655–666. 10.1093/plankt/fby039. 30487658 PMC6247811

[ref46] Yue M.; Yang M.; Zhang D.; Xiang D.; Hou Y.; Han J. Hybrid Au/ZnO Hexagonal Pyramid Nanostructures: Preferred Growth on the Apexes of the Basal Plane than on the Tip. J. Phys. Chem. C 2015, 119, 4199–4207. 10.1021/jp512570b.

