# Supplementary material for: ZnO Quantum Photoinitiators as an All-in-One Solution for Multifunctional Photopolymer Nanocomposites
Source: ACS Nano. 2023 Oct 3;17(20):20366–75. doi: 10.1021/acsnano.3c06518 (PMC10604079; doi:10.1021/acsnano.3c06518)

Supporting Information

ZnO Quantum Photoinitiators as an All-in-one Solution for Multifunctional Photopolymer Nanocomposites

Tom Naor, Shira Gigi, Nir Waiskopf, Gila Jacobi, Sivan Shoshani, Doron Kam, Shlomo Magdassi, Ehud Banin*, and Uri Banin**

Table of Content:

Figure S1: ZnO NRs synthesis progress

Figure S2: Swelling effect

Figure S3: ZnO NRs and DLP 385 nm projector optical characteristics

Figure S4: Mechanical properties of nanocomposites with different amounts of ZnO

Figure S5: Tensile test dog-bone specimen dimensions

Figure S1: ZnO NRs synthesis progress. TEM images and size statistics of aliquots that were taken from the crude during the synthesis after (a) 1 hour, (b) 2 hours, (c) 3 hours, (d) 4 hours, and (e) 5 hours at 60 °C. The scale bars represent 50 nm.

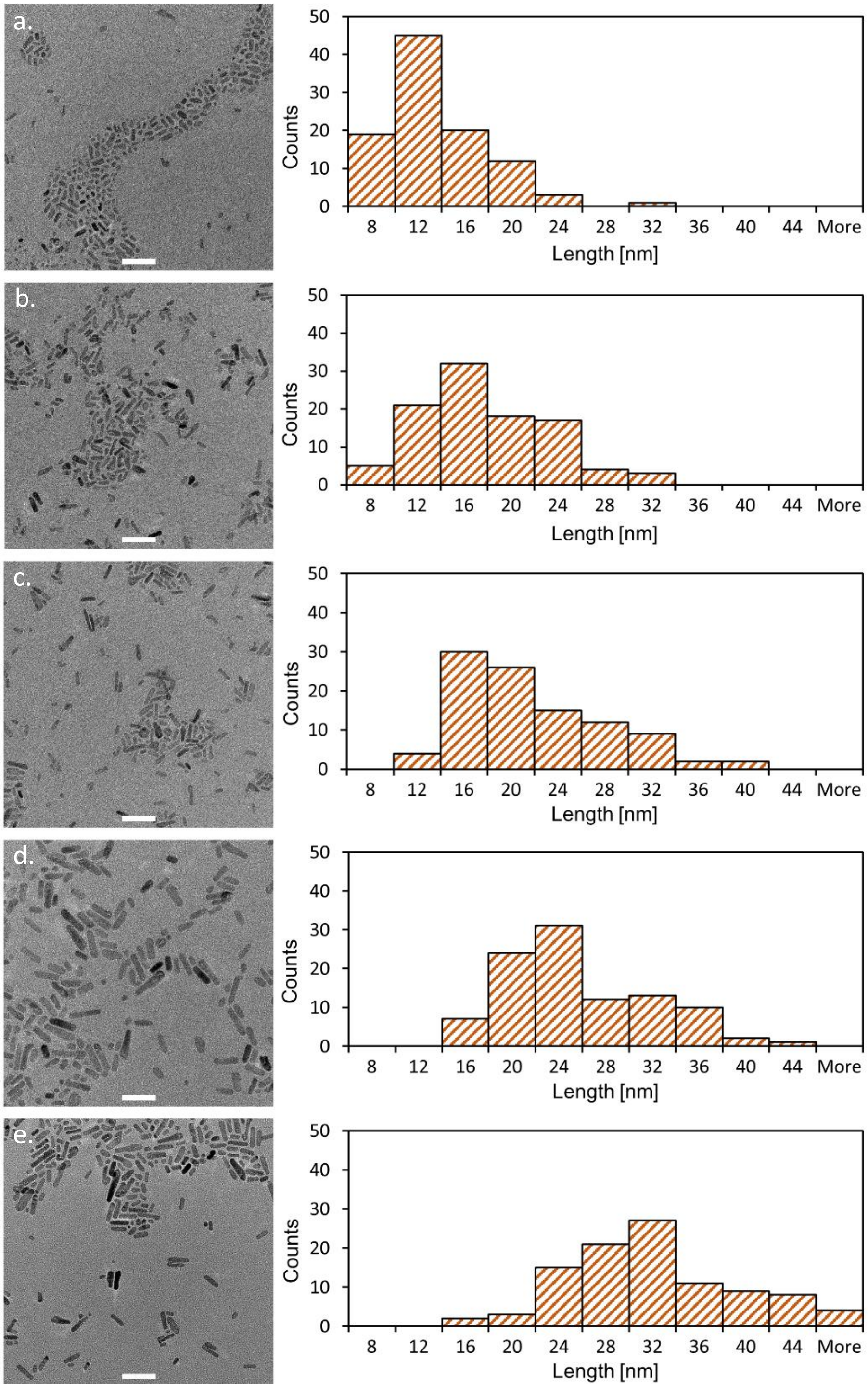

Figure S2: Swelling effect. Wet weight per dry weight of PEGDA hydrogels with different amounts of PEGDA in an aqueous solution. Comparing ZnO NRs acting as QPI (blue), reference system (red) with ZnO NRs only as a property modifier filler, and a control system (black) photoinitiated by TPO.

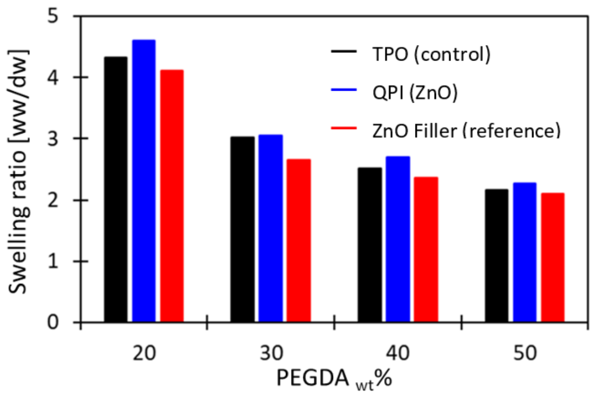

Figure S3: Normalized absorbance spectrum of ZnO NRs (red) and emission spectrum of the DLP printer (Black).

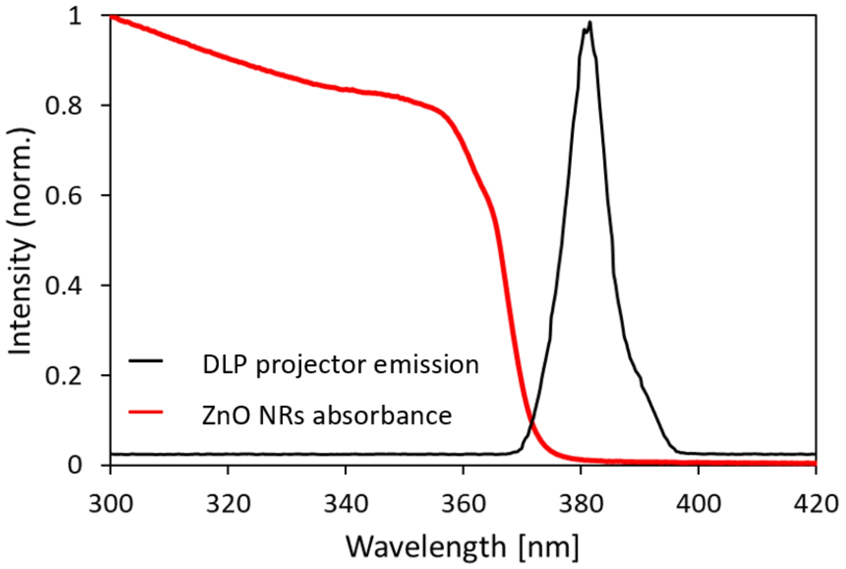

Figure S4: Summary of the mechanical properties of PEGDA hydrogel nanocomposites with different amounts of ZnO, under (a-c) dry and (d-f) wet conditions, comparing ZnO NRs acting as QPI (blue), and ZnO NRs as a property modifier filler (red).

Dry:

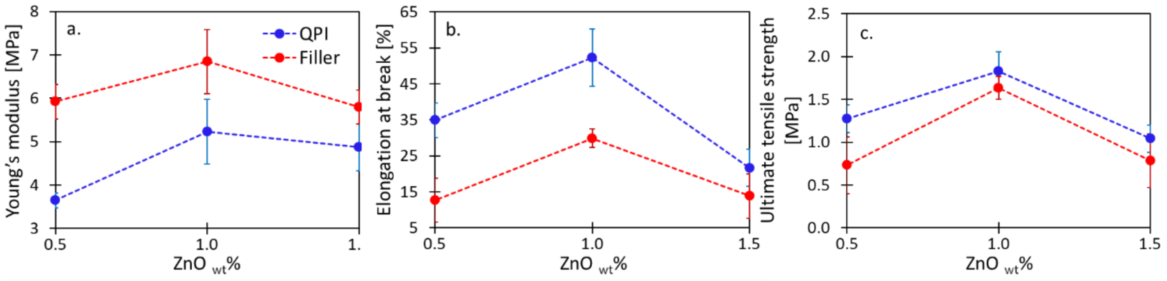

Wet:

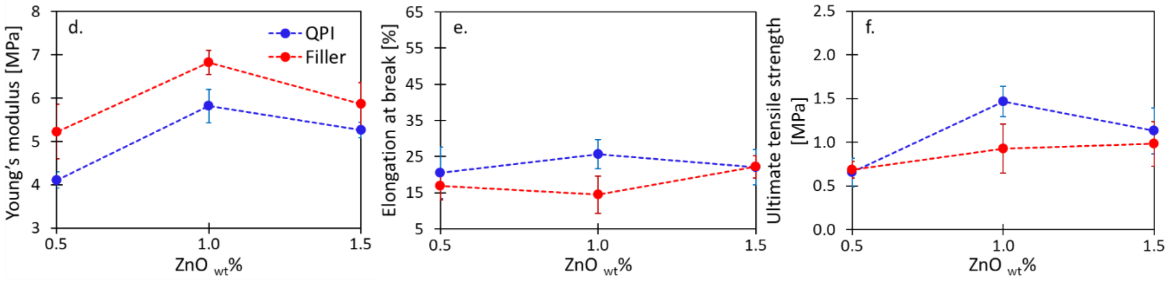

Figure S5: Tensile test dog-bone specimen dimensions.

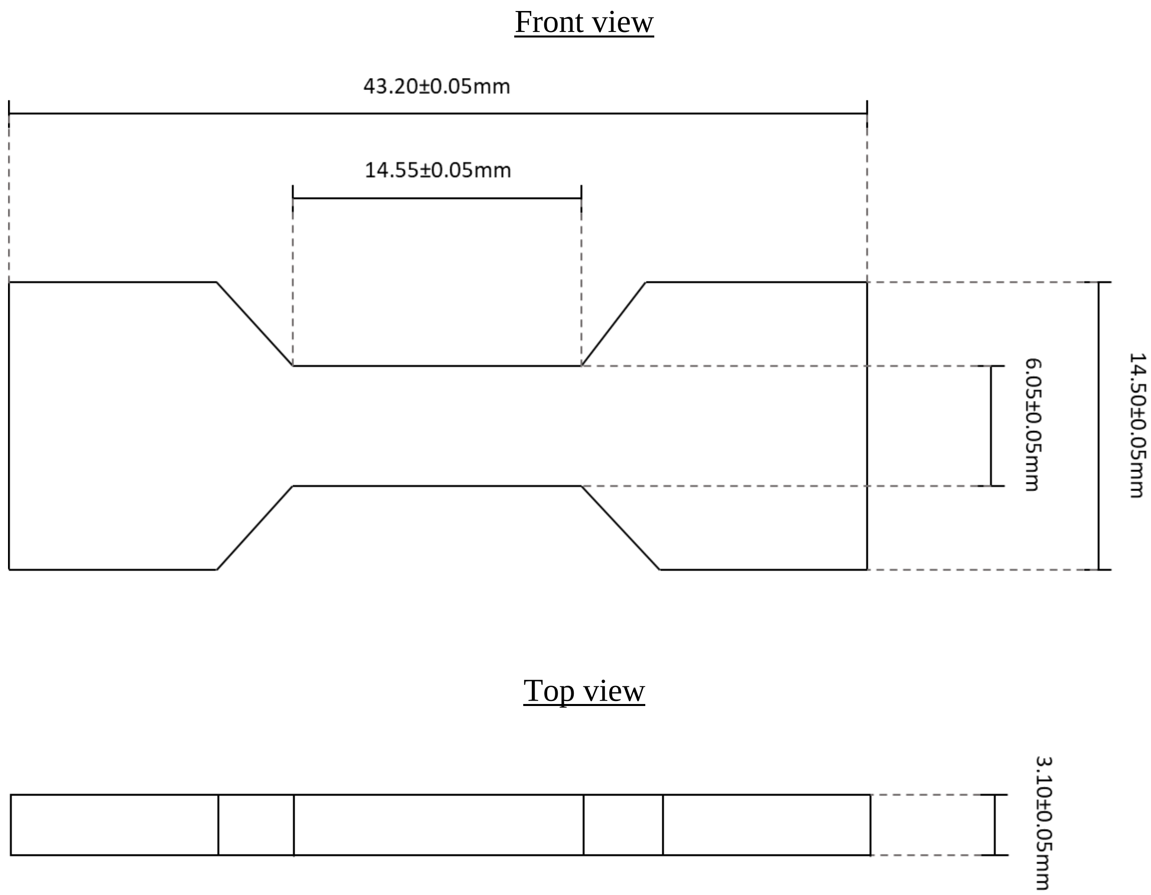

Supplement: Supplementary file 1 — nn3c06518_si_001.pdf [file nn3c06518_si_001.pdf]
